# Alteration of Coal Fly Ash Induced by Aging Treatment: Insights from Mineral Quantification Analysis

**DOI:** 10.1021/acsomega.5c06820

**Published:** 2025-10-10

**Authors:** Tsugumi Seki, Tatsuru Takahashi, Taiji Chida, Chihiro Inoue, Yasumasa Ogawa

**Affiliations:** 1 Department of Quantum Science and Energy Engineering, Graduate School of Engineering, 13101 Tohoku University, Aoba 6-6-01-2, Aramaki, Aoba, Sendai 980-8579, Japan; 2 Seafloor Mineral Resources Department, 50164 Japan Organization for Metals and Energy Security, 2-10-1 Toranomon, Minato-ku, Tokyo 105-0001, Japan; 3 Graduate School of Environmental Studies, 13101 Tohoku University, 6-6-20, Aoba, Aramaki, Aoba-ku, Sendai 980-8579, Japan; 4 Department of Earth Resource Engineering and Environmental Science, Faculty of International Resource Sciences, 12712 Akita University, 1-1, Tegatagakuen-machi, Akita 010-8502, Japan

## Abstract

The mineral composition of coal fly ash (FA) subjected to an aging treatment was analyzed using mineral liberation analysis (MLA) to elucidate the alteration behavior of FA, including changes in the leaching behavior of toxic elements such as B, As, and Se. Three types of FA generated from a coal-fired power plant in Japan, with initial pH values of 4, 9, and 12, were subjected to leaching experiments. These FA samples exhibited different alteration behaviors depending on their initial pH. FA1, with a pH of 12, exhibited a significant decrease in the leaching of B and Se after aging, and the MLA results revealed a slight change in the major mineral contents. However, the amount of ettringite, Ca_6_Al_2_(SO_4_)_3_(OH)_12_•26H_2_O, slightly increased during the aging period, which was consistent with the leaching behavior of Ca, SO_4_^2–^, and Al. In contrast, FA2, with an initial pH of 4, did not exhibit decreases in the leaching of B, As, or Se during aging, and the MLA results revealed a significant increase in quartz owing to the dissolution of aluminosilicates during aging. For FA3, with an initial pH of 9, the leaching of B and Se decreased slightly following aging, and the MLA results revealed a significant increase in quartz and mullite and a significant decrease in muscovite-like K-aluminosilicate. Furthermore, the amount of ettringite increased slightly, indicating the potential formation of monosulfate (kuzelite), Ca_4_Al_2_(OH)_12_(SO_4_)•6H_2_O, considering the stability of ettringite under the given pH conditions. These findings suggest that the immobilization of toxic elements during aging is influenced by the initial pH and secondary mineral phase formation. Moreover, the results offer valuable insights into the environmental alteration behavior of FA and demonstrate the applicability of MLA for characterizing heterogeneous environmental materials with complex compositions, such as FA.

## Graphical abstract

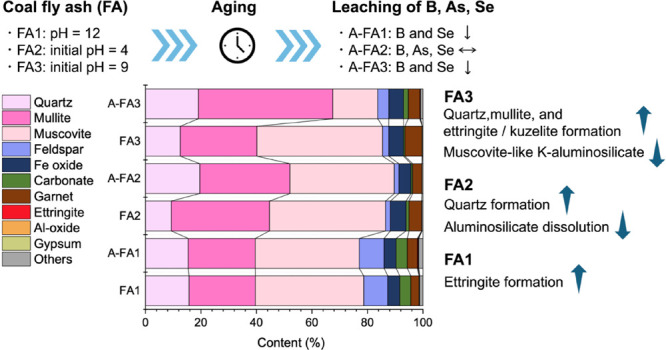

## 1 Introduction

Coal fly ash (FA) is a byproduct of the burning of pulverized coal in power plants and produced in large amounts worldwide. FA primarily consists of inorganic components, such as silica, alumina, iron oxide, and calcium oxide.^1^ FA has been widely used as a cement additive in recent years because of its beneficial properties, including long-term strength, durability, flowability, and low alkalinity, compared to ordinary Portland cement (OPC).^2–9^ Moreover, its use in construction materials contributes to cost reduction and has the potential to reduce CO_2_ emissions.^10–13^

However, FA contains toxic elements, such as B, As, and Se, thus raising concerns regarding groundwater and soil contamination if not handled properly.^14–16^ Various methods have been proposed to immobilize these toxic elements. For example, Tian et al. demonstrated that mixing FA with hydroxylated calcined dolomite (a mixture of Ca­(OH)_2_, Mg­(OH)_2,_ and MgO) effectively suppressed the leaching of B, Cr, As, Se, Mo, and W.^17^ Belviso studied the impact of zeolitization on the mobility of As, Cd, Cr, Se, and Pb.^18^ Seki et al. found that the leaching of As and Se was significantly suppressed by mixing FA with blast furnace cement.^19^ Moreover, Ogawa et al. proposed an aging treatment, that involved the mixing of FA with approximately 30 wt % water followed by 1 week of curing, as a cost-effective method to decrease the leaching of B, As, Cr, and F.^19–22^ Nevertheless, the effectiveness of the aging treatment varied depending on the FA sample because the FA composition is influenced by coal source and power plant conditions. In some cases, leaching of certain elements may even be accelerated, which is a serious problem.^19,22^ This aging process can also be regarded as an accelerated test for the alteration of FA when it absorbs moisture from the air or comes into contact with water during the cementation process. Therefore, understanding the alteration behavior of FA during the aging process is crucial for comprehending the leaching behavior of the toxic components in FA, which is essential for its safe and effective utilization.

Analyzing FA particle itself is a fundamental approach to better understanding the alteration behavior of FA. Various analytical methods have been employed to analyze FA particles. For instance, Hatori et al. employed microparticle-induced X-ray emission (PIXE) to observe individual FA particles and revealed a homogeneous distribution of trace elements, such as V, Zn, and As.^23^ Wang et al. used laser ablation inductively coupled plasma mass spectrometry (LA-ICP-MS) to study the distribution and enrichment of rare earth elements (REEs) into aluminosilicates, Ca-(Fe)-enriched aluminosilicates, Fe-oxides, and SiO_2_ phases in FA.^24^ Furthermore, Kutchko and Kim used scanning electron microscopy and energy dispersive X-ray spectroscopy (SEM-EDS) to analyze the surface and internal structures of FA particles and revealed that they were primarily composed of amorphous aluminosilicate and iron-rich spheres.^25^ However, these techniques require manual particle observation and data analysis; thus, analyzing a sufficient number of particles to obtain statistically significant data is labor-intensive and time-consuming. This is particularly challenging for samples containing trace elements or heterogeneous materials, where the workload is high and there are limitations to obtaining statistically significant results.

To address these issues and limitations, this study employed mineral liberation analysis (MLA), a technique traditionally used to characterize ore samples and optimize mining and mineral processing operations.^26–28^ MLA identifies the constituents of samples based on differences in grain boundaries and brightness in backscattered electron (BSE) images and elemental analyses by SEM-EDS, with references supplied by a mineral database.^26–29^ This enables the automatic simultaneous analysis of tens of thousands of particles per sample, thereby offering a more comprehensive understanding of the chemical properties of FA. By applying MLA to FA samples, this study intends to collect representative data for the entire sample and analyze small amounts of components, thus aiding in the investigation of alterations induced by the aging process.

Therefore, the objective of this study is to apply MLA to FA samples, investigate the alteration behavior during the aging process, and help elucidate the leaching and immobilization mechanisms of toxic components by combining these findings with a basic leaching experiment. In this study, three types of FA samples produced in Japan were selected, with a particular focus on toxic elements (B, As, and Se) that have frequently been reported to exceed the leaching regulation limits in Japan.

## 2 Material and Method

### 2.1 FA Samples and Characterization

Three FA samples, referred to as FA1, FA2, and FA3, were collected from a power plant in Japan that use a steam power generation method and a pulverized coal-fired boiler. The chemical composition was determined by energy-dispersive X-ray fluorescence spectroscopy (XRF, Epsilon 5, Malvern Panalytical) and is listed in Table 1. The samples were classified as class-F according to ASTM C618–19.^30^ Note that the data of B content cannot be measured by energy-dispersive XRF. Instead, it was determined by analyzing the B concentration in the liquid phase obtained from the extraction test with 1 mol/L HCl by using ICP-OES (Agilent 5100, Agilent Technologies). The particle size distributions were measured using a laser particle size analyzer (MASTERSIZER 3000, Malvern PANalytical), as shown in Figure 1. The modal diameters of FA1, FA2, and FA3 were 48.6 μm, 25.7 μm, and 42.8 μm, respectively.

**Table 1:** Chemical Composition of the FA Samples

(%)	FA1	FA2	FA3
SiO_2_	62.32	60.92	62.91
Al_2_O_3_	21.94	24.63	23.96
CaO	3.47	1.32	1.41
MgO	1.13	0.97	1.13
Na_2_O	1.03	0.33	0.60
K_2_O	1.72	1.35	1.61
SO_3_	0.75	1.14	0.72
TiO_2_	1.33	1.56	1.64
P_2_O_5_	0.00	0.33	0.05
Fe_2_O_3_	5.87	4.92	5.48

(mg/kg)			
B	174.3	80.0	36.3
As	4.5	30.9	57.7
Se	2.4	6.2	4.3

**Figure 1:**
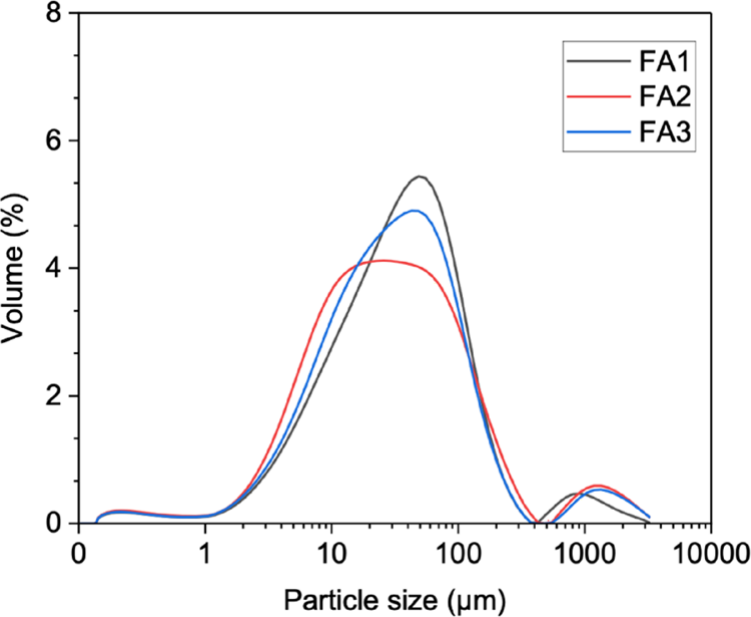
Particle size distribution of the FA samples.

### 2.2 Leaching Experiment

The leaching experiment was conducted according to JLT-18 (Ministry of the Environment of Japan, Notification No.18, 2003). All experiments were conducted in triplicate to confirm reproducibility. Three-gram FA samples were mixed with 30 mL of ultrapure water (the ratio of liquid and solid phase (L/S)=10) in a 50 mL centrifuge tube (polypropylene) and shaken at 200 rpm for 6 h at 20°C. In addition, changes in pH and the concentrations of the components over 1, 3, 10, 40, 120, and 360 min were also obtained to track the leaching behavior. After shaking, the samples were centrifuged at 3300 rpm for 20 min and filtered through a 0.45 μm syringe filter. The pH of the filtrates was measured using a pH meter (LAQUA, HORIBA), and the concentration of each component in the filtrates was quantified using ICP-MS (NexION 300, PerkinElmer) and ICP-OES (Agilent 5100, Agilent Technologies). X-ray diffraction (XRD) patterns of the solid phases were obtained using an X-ray diffractometer (Miniflex, Rigaku) after air-drying at room temperature for a week. The surfaces of the solid-phase particles were analyzed using SEM-EDS (SU6600, Hitachi High-Tech and QUANTAX XFlash 5060FQ+XFlash6|10, Bruker).

### 2.3 Aging Treatment

The aging treatment was based on a report by Ogawa et al.^20^ Three-gram of FA samples were mixed with 1.2 mL of ultrapure water in a 50 mL centrifuge tube (polypropylene), and the resulting paste (28.6% water content) was capped and aged without shaking at 20°C in the dark for a week. After the aging period, an additional 28.8 mL of ultrapure water was added to a total volume of 30 mL (L/S=10) for the leaching experiment. The subsequent leaching experiment was performed as described in 2.2. Hereafter, the aged samples are referred to as A-FA1, A-FA2, and A-FA3.

### 2.4 Mineral Liberation Analysis

The compositions of the constituent minerals were analyzed using an MLA system (MLA650, FEI Company) equipped with SEM-EDS (QUANTA 650, FEI Company and XFlash6|30, Bruker), owned by the Japan Organization for Metals and Energy Security (JOGMEC). For the MLA, the samples for measurement were prepared by embedding FA in resin, followed by grinding, polishing, and carbon coating.

#### 2.4.1 Preparation of Resin-Embedded Samples

For resin embedding of FA samples, 0.5 g of FA and 0.15 g of carbon powder were mixed in a cylindrical plastic cup, followed by the addition of 0.5 to 1.0 mL of ethanol, which was then mixed thoroughly. The mixture was sonicated for 5 min in an ultrasonic bath to enhance mixing and dispersion. The main resin and hardener were premixed separately in a paper cup at a volume ratio of 15:2. Subsequently, 5 mL of the resin mixture was poured into the plastic cup containing the sample, stirred with a spoon, and sonicated again for 5 min. To remove air bubbles, the mixture was placed in a vacuum desiccator for 30 min with repeated cycles of air intake and exhaust. The resin was then cured in an oven at 35°C for 4 h. Once solidified, the sample was cut lengthwise using a diamond cutting device. The cross sections were polished with silicon carbide polishing sheets to achieve a smooth surface, washed with ethanol, and dried. The specimens were placed face down in a silicone mold, and resin was poured over them until they were fully immersed. The mold was subjected to additional defoaming in a vacuum desiccator with repeated cycles of air intake and exhaust, followed by curing in an oven at 35 °C for an additional 4 h.

After curing, the resin was removed from the mold. An image of the resin-embedded samples is shown in Figure 2. The observation surfaces of the resin samples were polished with #800, #1000, and #1200 grit abrasive papers using an automatic polishing machine (DIGIPREP301, Metkon) followed by polishing with 3 μm and 1 μm diamond suspensions. Finally, the observation surface was coated with carbon using a vacuum evaporation system.

**Figure 2:**
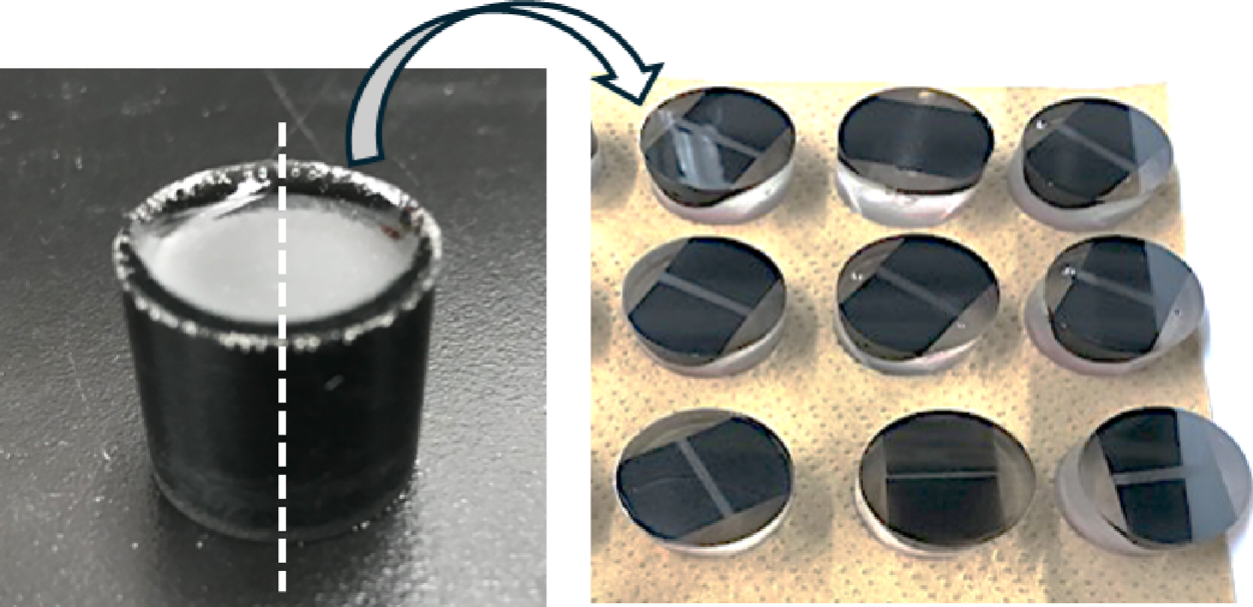
Image of the resin embedded samples.

#### 2.4.2 Sample Analysis

Mineral analysis was performed in GXMAP mode, in which multiple beams are irradiated onto a single particle (Figure 3(a)), rather than XBSE mode, in which a single beam is irradiated onto a single grain. GXMAP mode is particularly suitable for analyzing samples such as FA, where the elements are heterogeneously distributed.^31,32^ For the analysis, a BSE image was first captured (referred to as frames in Figure 3(b)), and EDS analysis was then performed on the particles within the acquired frame. This process was repeated by moving to the next frame, capturing a BSE image, and performing EDS analysis until the specified number of frames, particles, or analysis time was reached. The mineral components were identified by comparing the EDS peaks with the mineral database in the MLA software (minerals with similar compositions were classified by manually filtering in the elemental content range), and mineral mapping images were generated (Figure 3(c)).

**Figure 3:**
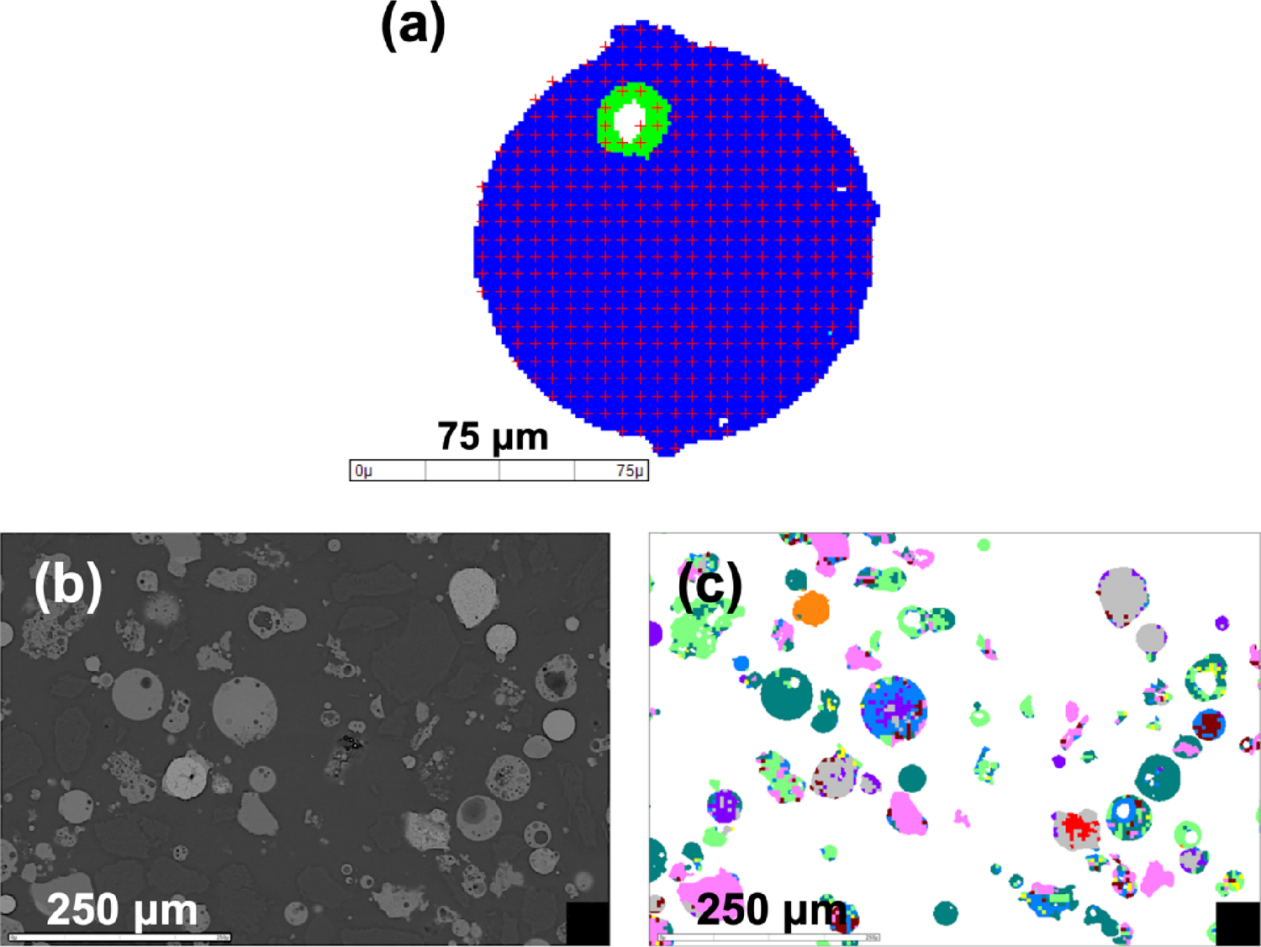
(a) Analysis spot of a FA particle in GXMAP mode (red marks indicate the position of electron beam irradiation), (b) BSE image, and (c) mineral mapping image of MLA.

The detailed measurement conditions are listed in Table 2. The pixel size was 1.5 μm, and the minimum grain size was 10 pixels. This means that grains of 2 × 5 pixels (= 3.0 × 7.5 μm) can be detected, but fine particles with diameters of a few micrometers observed in the SEM image (Figure 9) were not analyzed. However, BSE observations with MLA clearly indicated that particles smaller than 10 pixels accounted for an extremely small proportion of the total sample weight. Therefore, excluding these particles from the analysis is not expected to significantly affect the overall analytical results. Each sample was measured once, and the number of analyzed particles is summarized in Table 3, which shows that more than several tens of thousands of particles were measured for all samples. Herein, the term ″particle″ refers to a discrete unit observed at the sample scale, such as FA particles or agglomerate, while the term ″grain″ refers to a single crystalline domain within a particle, characterized by its own crystal orientation and boundaries. For instance, Figure 3(a) shows one particle consisting of two grains (green and blue).

**Table 2:** MLA Measurement Conditions For FA

conditions	
mode	GXMAP
voltage	25 kV
probe current	40 μm
BSE calibration	Cu
resolution	1000 × 1000 px
pixel size	1.5 μm/px
quartz EDX count	300–400 kcps
step size	2 px
minimum grain size	10 px
magnification	276
frame number	168

**Table 3:** Number of Measured Particles for Each Sample

particle counts	
FA1	81,334
A-FA1	77,529
FA2	65,473
A-FA2	67,372
FA3	84,754
A-FA3	43,341

## 3 Results

### 3.1 XRD Patterns

Figure 4 shows the XRD patterns of the FA samples before and after the aging treatment. Quartz (SiO_2_) and mullite (3Al_2_O_3_•2SiO_2_), the primary constituent minerals of FA, were identified in all samples. The broadening observed between 5° and 35° is attributed to the presence of amorphous compounds. Jankowski et al. reported that FA samples from an Australian coal-fired power plant contained 40–80% amorphous compounds, as determined by XRD and Rietveld analysis.^33^ Bakharev and Halder et al. also identified hematite via XRD analysis of Class-F FA.^34,35^ In contrast, hematite and other minerals were not detected in this study, possibly due to poor crystallinity or peak overlap with the major phases in the XRD patterns.

**Figure 4:**
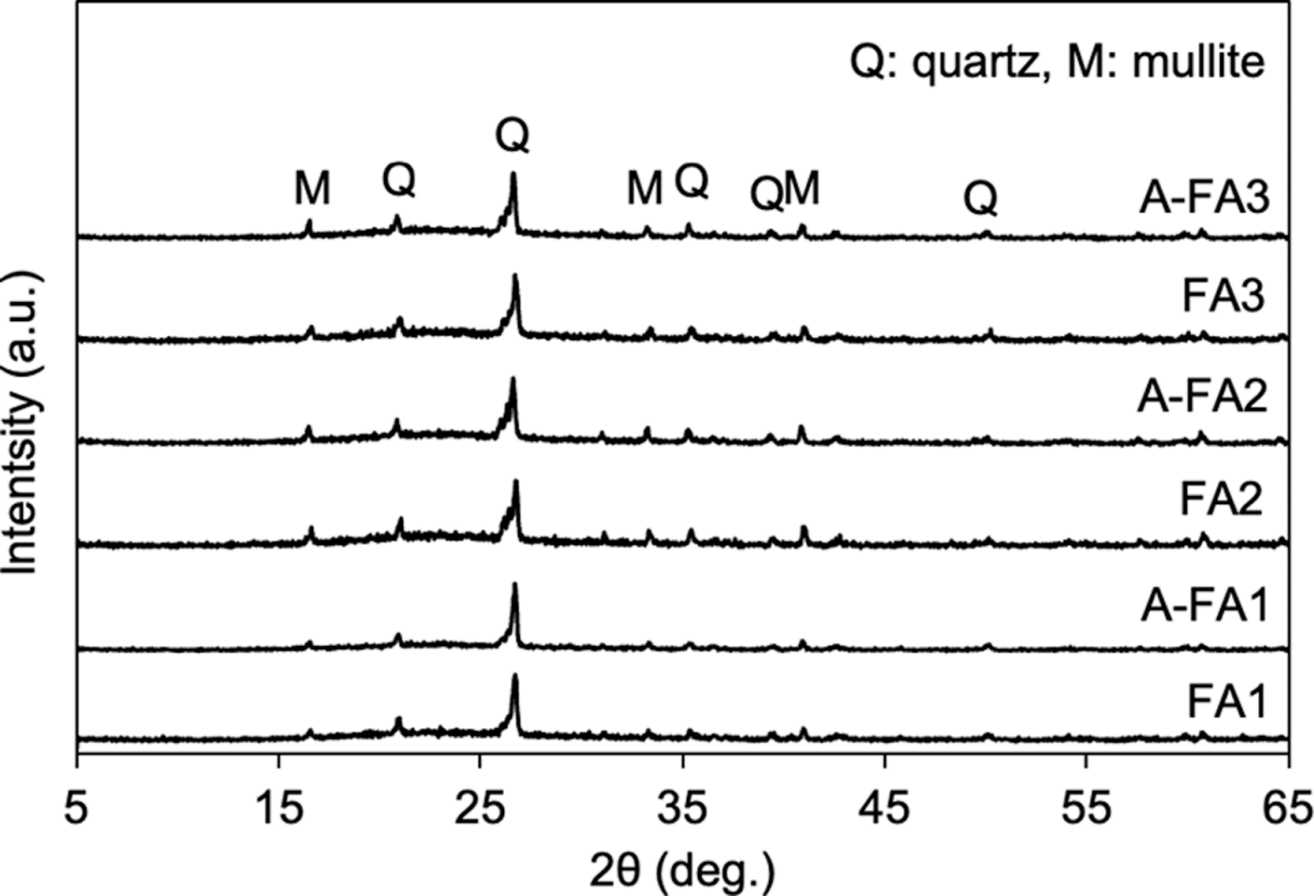
XRD patterns of the samples before and after the aging treatment.

### 3.2 pH and Leaching Amounts of B, As, and Se

The pH and leaching concentrations of B, As, and Se are shown in Figure 5, with error bars representing the standard error from triplicate experiments. The pH of FA1 was higher than that of FA2 and FA3, which can be attributed to its relatively higher content of alkaline components, particularly the CaO content.^16,36–43^ The pH did not change significantly before or after the aging treatment in any of the samples.

**Figure 5:**
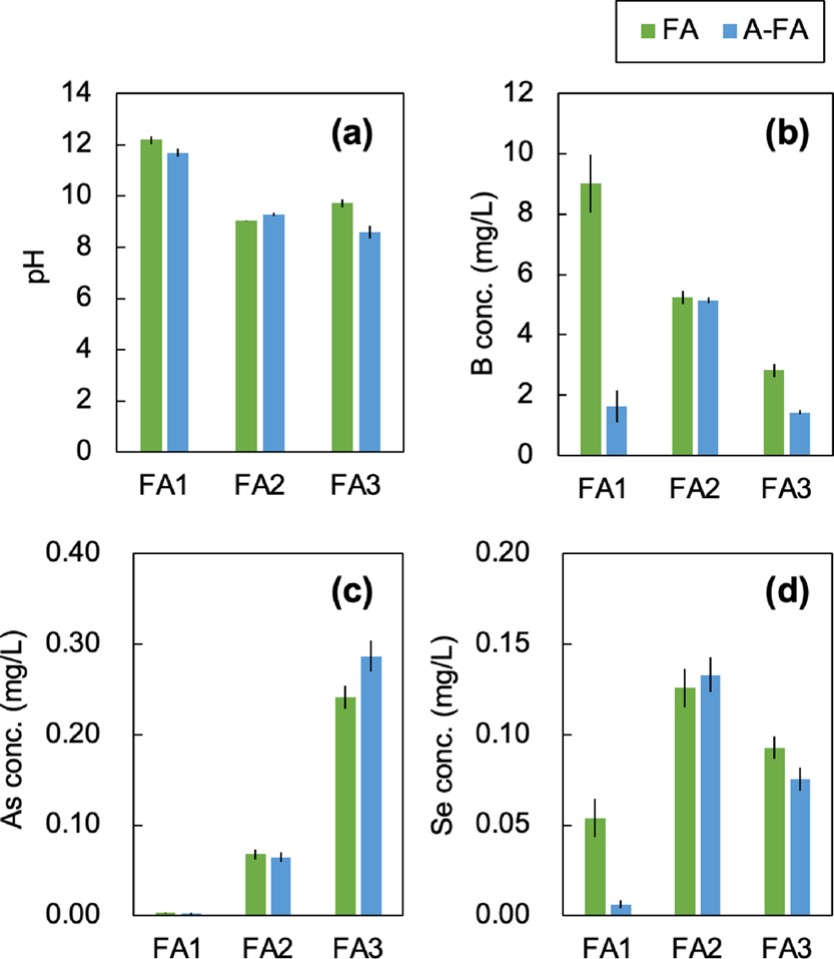
(a) pH and leaching concentrations of (b) B, (c) As, and (d) Se before and after the aging treatment. Error bars represent the standard error of triplicate experiments.

Regarding the leaching concentrations of B, As, and Se, the concentrations of B and Se in FA1 after aging decreased markedly, from 9.0 mg/L to 1.6 mg/L for B and from 0.054 mg/L to 0.006 mg/L for Se. Similarly, the leaching concentrations of B and Se in FA3 also decreased after aging. In FA1, the As concentration remained very low (0.003 mg/L), even before aging, while in FA3, it slightly increased with aging. In contrast, in FA2, leaching of B, As, and Se was not affected by aging.

### 3.3 pH and Leaching Behavior of Each Component with Time

Temporal changes in the pH and component concentrations during the 6-h leaching experiment are shown in Figures 6–8. Expanded data for the initial 40 min are shown in Figures S1–S3. The concentrations of Si in FA1, Mg in both FA1 and A-FA1, and Fe in all samples were below the detection limit and are therefore not displayed here.

**Figure 6:**
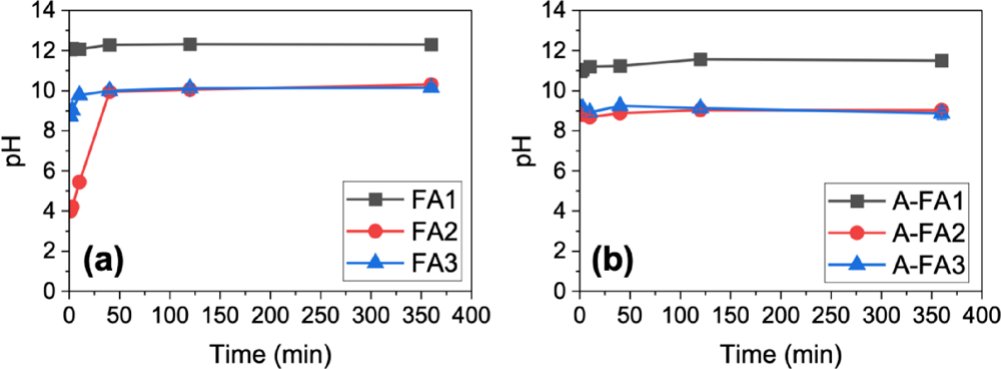
pH of the leachates with time during the 6 h leaching experiment. Error bars represent the standard error of triplicate experiments.

**Figure 7:**
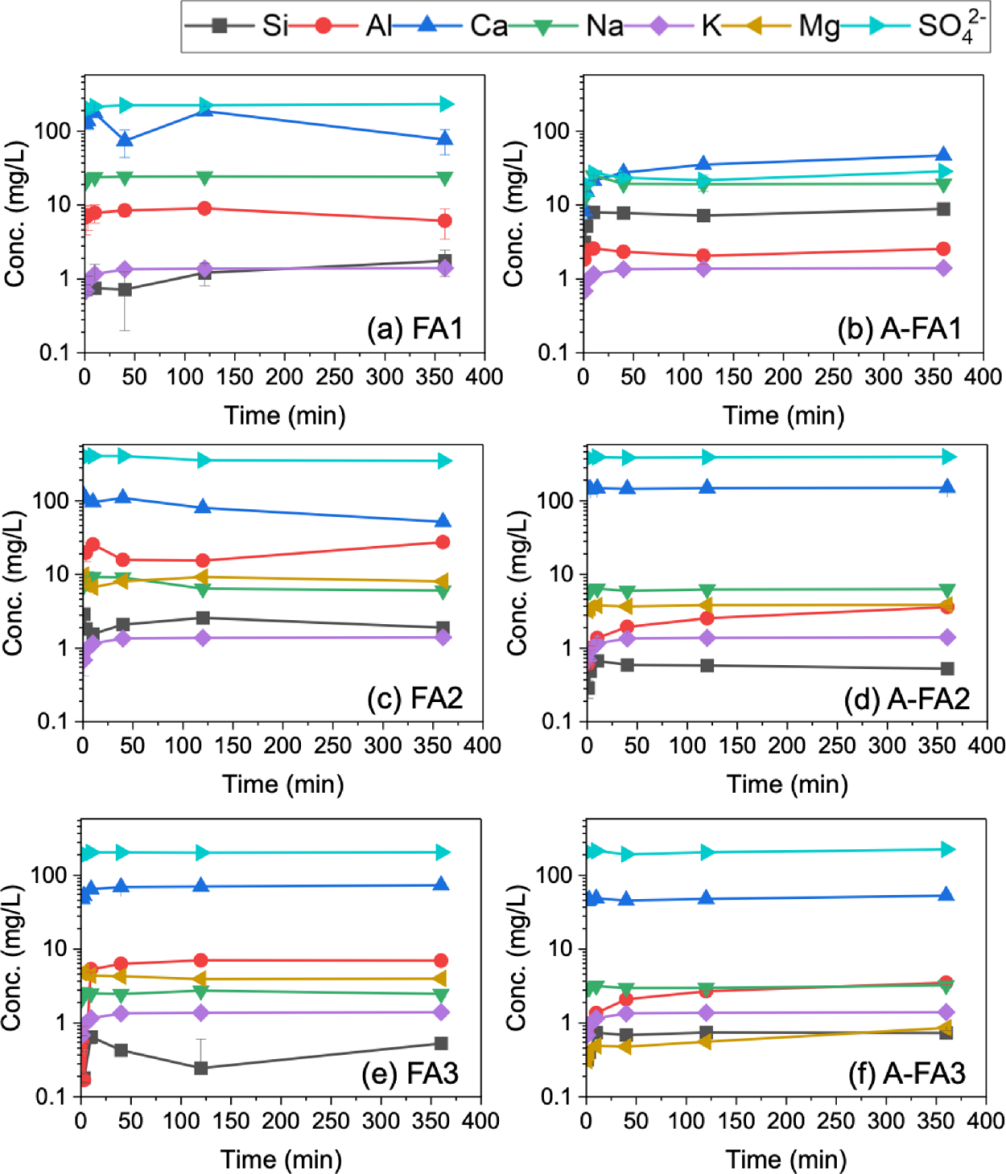
Leaching behavior of major components (Si, Al, Ca, Na, K, Mg, and SO_4_^2–^) during the 6 h leaching experiment. Note that the concentrations of Si from FA1, Mg from FA1 and A-FA1, and Fe from all samples were below the quantification limit and not included. Error bars represent the standard error of triplicate experiments.

**Figure 8:**
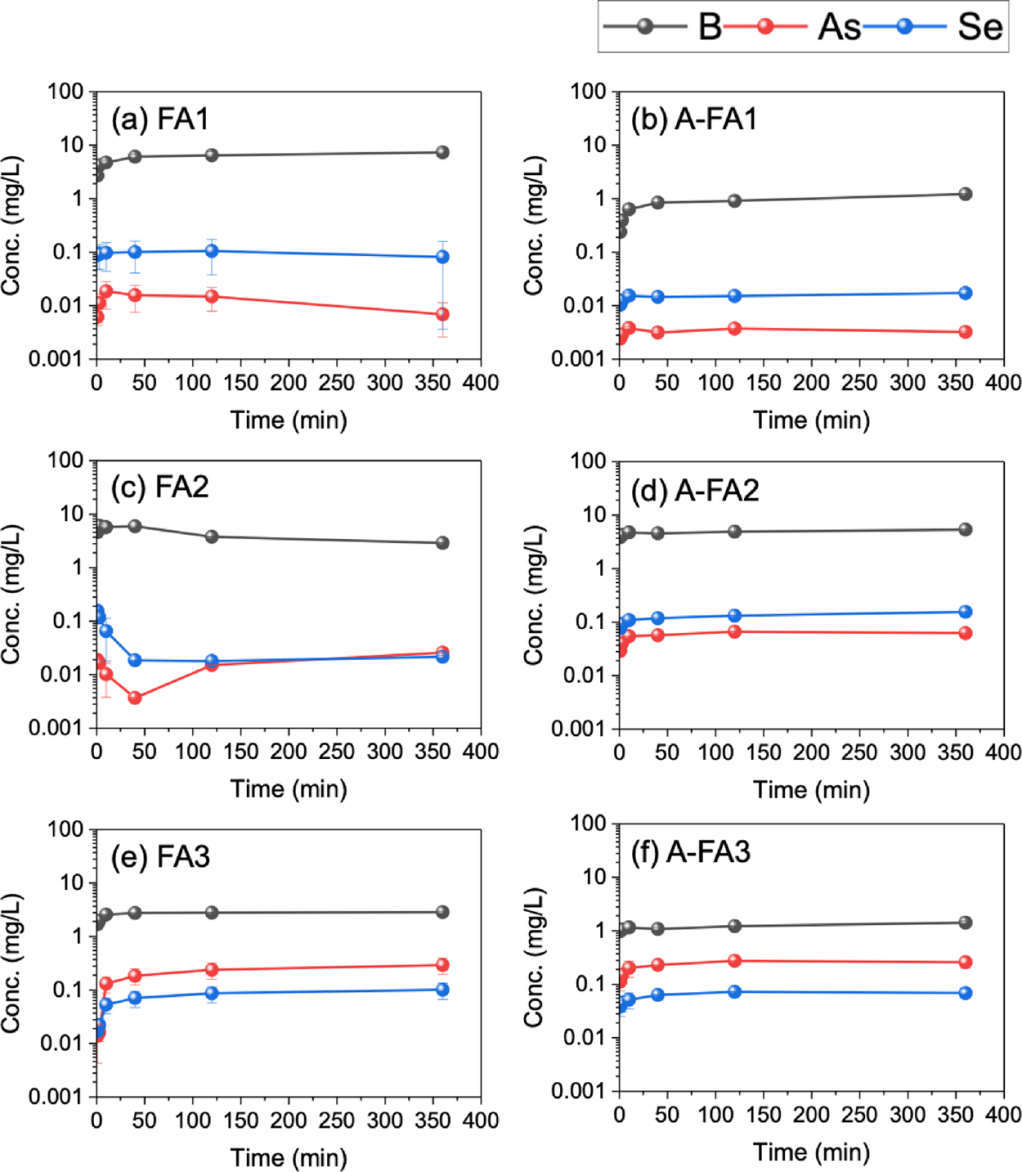
Leaching behavior of toxic elements (B, As, and Se) during the 6 h leaching experiment. Error bars represent the standard error of triplicate experiments.

As shown in Figure 6, the pH of the leachates reached a steady state after 40 min to 2 h for all samples. The initial pH (at 1 min) was pH 12.2 for FA1 (highly alkaline), pH 4.0 for FA2 (acidic), and pH 8.7 for FA3 (weakly alkaline). After 40 min, the pH of FA2 and FA3 increased to 9.9 and 10.0, respectively. The differences in pH among these samples can be attributed to the composition of the samples, as mentioned in Section 3.2. In particular, the lower pH observed in FA2 is attributable to its lower CaO content and relatively high SO_3_ content.

For each component, it is noteworthy to consider the behavior of the changes in Al concentration, which decreased with aging treatment in all samples. Moreover, changes in the Al concentration during the 6-h leaching experiment varied for each sample. For instance, the Al concentration in FA1 was initially the highest and then decreased after 2 h. In FA3, the Al concentration gradually increased initially. Such behavior of Al, which is instantaneously released upon contact with water (initially higher concentration) is derived from the surface-bound components of FA, while the Al released over time originates from the dissolution or dispersion of the glassy particles.^44^ The decrease in Al concentration over time was likely due to adsorption or precipitation on the surface of the FA particles. Additionally, as shown in Figure 8, the concentrations of As and Se varied in harmony with the dissolution and precipitation of Al. This is consistent with our previous study, in which the glass component in FA was shown to play a significant role in the leaching of B, As, and Se.^42^

Regarding the concentration of Ca, the aging treatment led to a significant decrease in FA1 and a slight decrease in FA3. Similarly, the concentration of SO_4_^2–^ in the leachate from FA1 decreased after the aging treatment. The decrease in Al concentration due to aging in FA1, along with the changes in Ca and SO_4_^2–^ concentrations, suggest that ettringite (Ca_6_Al_2_(SO_4_)_3_(OH)_12_•26H_2_O) may have formed during the aging process. Moreover, the leaching behavior of B correlates with the concentration of Ca, suggesting that B may be incorporated into Ca-bearing compounds such as ettringite.^45,46^

### 3.4 SEM-EDS Observation

SEM-EDS images of FA1 and A-FA1 are shown in Figure 9, and those of the other samples are shown in Figure S4. The typical spherical morphology of the FA particles was consistent with that in a previous study.^25^ After aging, rough particle surfaces and aggregates of fine particles were observed, indicating the formation of precipitates on the particle surfaces during the aging period.

In the EDS analysis, no peaks corresponding to B, As, or Se were observed. In the EDS mapping images, Si and Al were distributed throughout the particles, and Fe was also present across the particles. Notably, Ca and S colocalized in specific areas, particularly after the aging treatment. These areas correspond to the locations of needle-like precipitates, suggesting the formation of ettringite during aging. Given that ettringite is well-known to form as needle-like crystals and is stable at pH above 10.5 to 11,^47–49^ this interpretation is further supported by the temporal trends observed in the pH and Ca and SO_4_^2–^ concentrations (Figures 6 and 7). In addition, the EDS mapping of A-FA2 (Figure S4­(b)) also showed needle-like crystals with Ca and S distribution. However, this result is inconsistent with the MLA results described in Section 3.5, suggesting that the formation of these structures may have been limited. In the mapping of A-FA3 (Figure S4­(c)), Ca and S were distributed in areas corresponding to the faceted tabular crystals. These distributions suggest the possible formation of monosulfate (kuzelite), Ca_4_Al_2_(OH)_12_(SO_4_)•6H_2_O, which has a platy, layered structure and a composition similar to that of ettringite (Ca_6_Al_2_(SO_4_)_3_(OH)_12_•26H_2_O).^50–52^ Although gypsum (CaSO_4_) also forms as needle-like or faceted tabular crystals, it was not identified by the MLA (Section 3.5).

In contrast, SEM-EDS analysis showed unique compositional differences for each observed particle, even within the same sample. Because the number of particles that can be analyzed using SEM-EDS is limited, obtaining data representative of the entire sample is challenging. Consequently, it was difficult to evaluate the differences in alteration behavior among FA1, FA2, and FA3, as well as the corresponding variations in the immobilization effect of toxic elements, based on SEM-EDS analysis alone.

**Figure 9:**
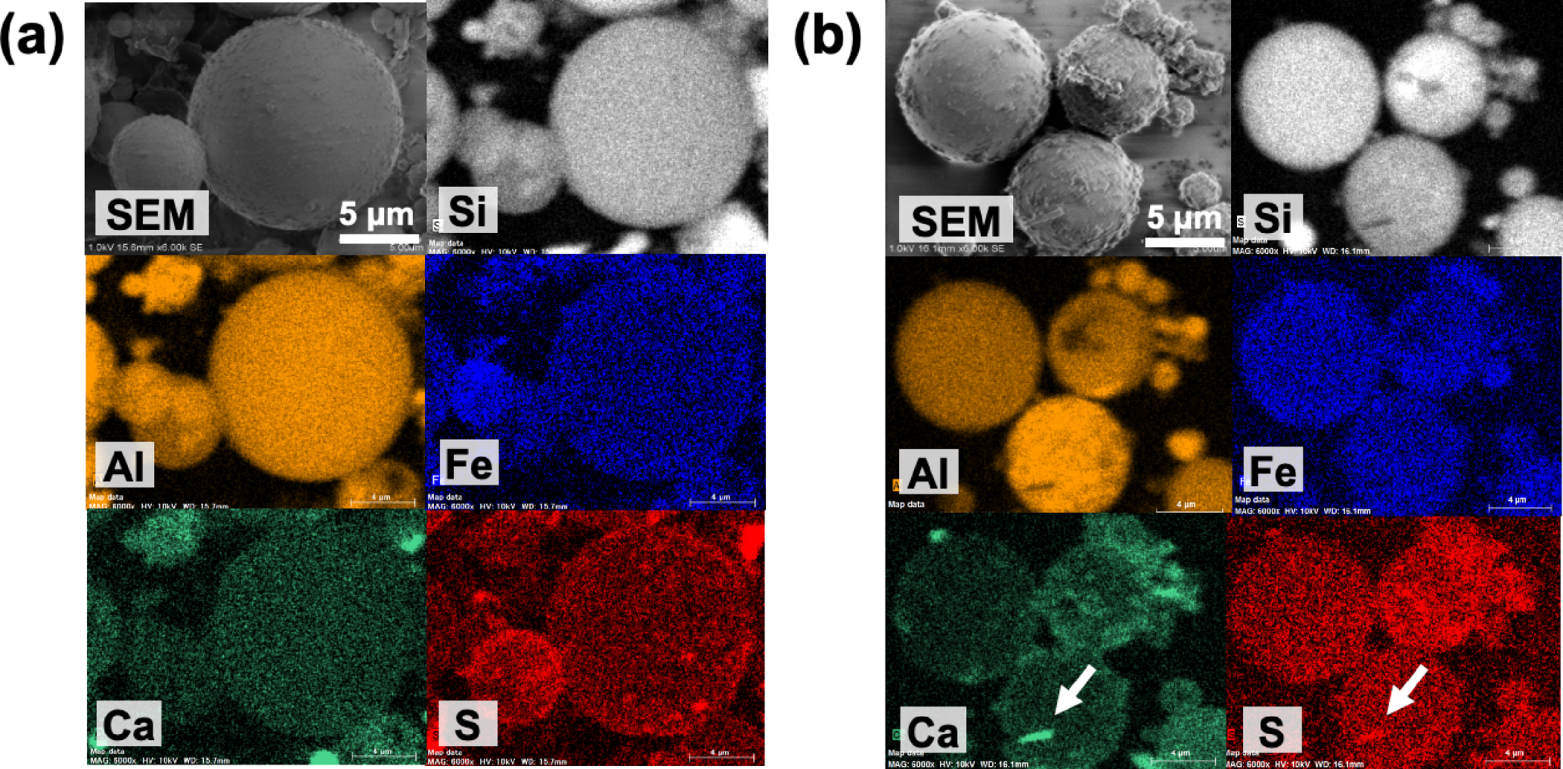
SEM-EDS images of (a) FA1 and (b) A-FA1 particles.

### 3.5 MLA Analysis

To enable a more macroscopic analysis, MLA was applied observe the FA particles. The minerals detected in the FA samples and their corresponding chemical formulas are listed in Table 4. It should be noted that, in MLA, the observed grains are referenced in the MLA database and systematically classified as ″minerals″ regardless of their crystallinity. For this reason, the quantified results of “mineral composition” should be interpreted as “compound composition” in the context of FA analysis, and their crystallinity should be discussed in conjunction with the results of the XRD analysis. For example, in Table 4 and Figures 10 and 11, mullite refers to glassy aluminosilicates, and muscovite refers to glassy K-aluminosilicates. Additionally, as shown in Table 4, the minerals from quartz to gypsum were considered the minerals of interest and were not assigned to any group, whereas the others were grouped to summarize the results.

**Table 4:** Minerals and Their Formulae Identified in the FA Samples by the MLA

group	mineral	formula
quartz		SiO_2_
mullite		Al_6_Si_2_O_13_
muscovite		KAl_3_Si_3_O_10_(OH)_1.9_F_0.1_
Al-oxide		Al_2_O_3_
garnet		(Fe(II), Ca)_3_(Al, Fe(III))_2_(SiO_4_)_3_
ettringite		Ca_6_Al_2_(SO_4_)_3_(OH)_12_
gypsum		CaSO_4_
feldspar	K-feldspar	KAlSi_3_O_8_
	albite	NaAlSi_3_O_8_
	anorthite	CaAl_2_Si_2_O_8_
	plagioclase	Na_0.5_Ca_0.5_Si_3_AlO_8_
Fe oxide	hematite	Fe(III)_2_O_3_
	magnetite	Fe(II)Fe(III)_2_O_4_
	ferrosilite	Fe(II)MgSi_2_O_6_
carbonate	calcite	CaCO_3_
	ankerite	Ca(Fe,Mg,Mn)(CO_3_)_2_
	magnesite	MgCO_3_
	siderite	FeCO_3_
	Mn-siderite	(Fe,Mn)CO_3_
others	apatite	Ca_5_(PO_4_)_3_(F,Cl,OH)
	rutile	TiO_2_
	actinolite (amphibole)	Ca_2_(Mg,Fe)_5_Si_8_O_22_(OH)_2_
	barite	BaSO_4_

**Figure 10:**
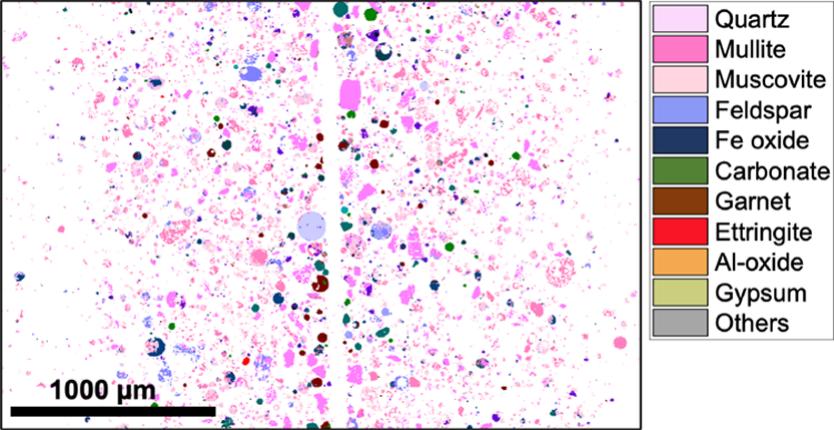
MLA mapping images of FA1. Note that the mineral names listed in these legends include amorphous phases.

**Figure 11:**
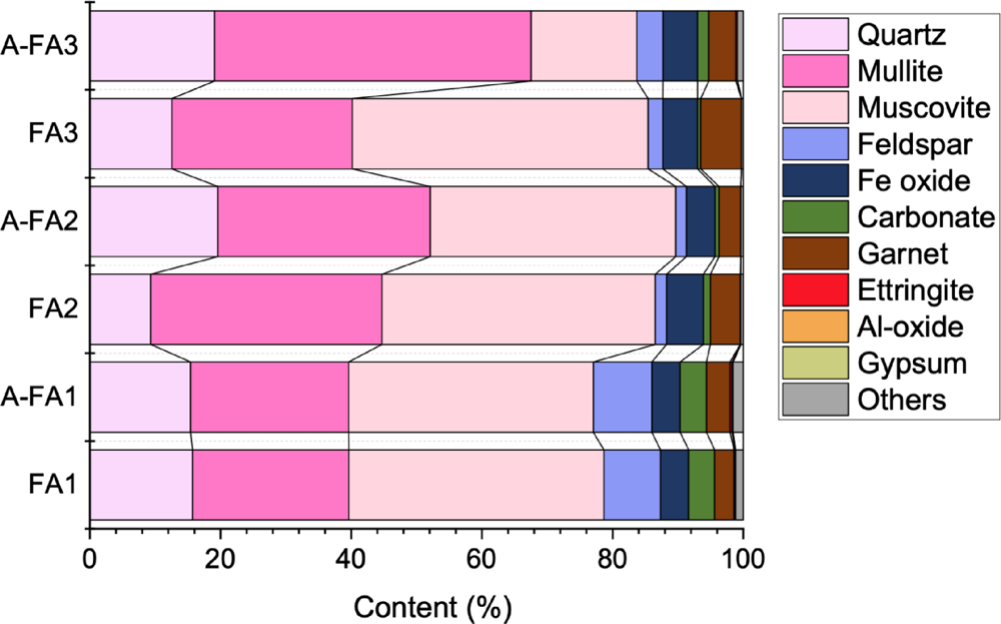
Compound compositions quantified by MLA. Note that the mineral names listed in these legends include amorphous phases.

An MLA mapping image of FA1 is shown in Figure 10, and those of the other samples are shown in Figure S5. All samples exhibited similar imaging characteristics, with some particles composed of multiple mineral phases and some showing hollow structures. Such hollow structures were attributed to the fact that the internal cross sections of the particles, rather than their external surfaces, were analyzed in this measurement because of the resin embedding and polishing steps applied during sample preparation.

The quantified results are shown in Figure 11. Notably, the aging treatment led to varying changes in the amount of quartz (SiO_2_), mullite (aluminosilicate), and muscovite (K-aluminosilicate) across the samples. The amounts of quartz in FA2 increased significantly after aging. In FA3, the quartz and mullite contents increased markedly while the muscovite content decreased substantially. In contrast, FA1 showed no significant change in the amounts of quartz and mullite during the aging treatment. These results suggest that FA1, FA2, and FA3 exhibit different dissolution and reprecipitation behavior of silica and aluminosilicates. Although the pH values of the leachates at 6 h were almost the same between FA2 and FA3 (Figure 5(a)), their alteration behaviors differed. This suggests that the initial pH (e.g., within 1–40 min, shown in Figure S1) may have a significant effect on these alteration behaviors, as explained in section 4.1.

The quantitative results for the minor phases such as ettringite, gypsum, calcite, and Al oxide are summarized in Table 5. The amount of ettringite increased in FA1 and FA3 but decreased in FA2 after aging. Gypsum was barely detected in any of the samples, suggesting that the Ca- and S-containing grains observed in the SEM-EDS images (Figures 9 and S4) could be classified as ettringite. The statistical significance of the changes in the amount of ettringite was evaluated using a two-proportion Z-test. In FA1, the ettringite content increased significantly from 0.15% to 0.29% by aging (Z = 5.98, *p* < 0.00001). Similarly, in FA3, the ettringite content showed highly significant increases from 0.03% to 0.24% by aging (Z = 11.24, *p* < 0.00001). Although Myneni et al. reported that ettringite can form even at pH values of 9.3 and 10.3,^53^ it is typically stable at pH above 10.5. Therefore, monosulfate (kuzelite) rather than ettringite likely formed in FA3, as indicated by the SEM-EDS observations. Monosulfate data were not present in the MLA database; therefore, the phases of monosulfate and ettringite, which have similar chemical compositions, were not differentiated and may have been collectively identified as ettringite. The current mineral database for cementitious materials is insufficient, and expanding the database represents an area for future research.

**Table 5:** Quantitative Results for Ettringite, Gypsum, Calcite, and Al Oxide

(%)	FA1	A-FA1	FA2	A-FA2	FA3	A-FA3
ettringite	0.15	0.29	0.06	0.03	0.03	0.24
gypsum	0.01	0.00	0.00	0.00	0.00	0.00
calcite	0.02	0.06	0.01	0.00	0.00	0.03
Al oxide	0.08	0.18	0.00	0.00	0.00	0.01

With respect to the uncertainty of MLA, some particles may have been lost during the resin embedding and polishing processes for sample preparation, and the resulting uncertainty is likely to have a greater impact than instrumental error. Furthermore, as mentioned in Section 2.4.2, even if the number of particles below the detection limit appears visually very small, these may still contribute to the overall analytical uncertainty. As confirmed by the Z-test mentioned above, the change in ettringite content before and after aging was statistically significant. However, considering these uncertainty factors, trace mineral phases that may be locally unevenly distributed in heterogeneous samples like FA should be regarded as semiquantitative. Improving sample preparation methods for fragile samples such as FA and enhancing quantification accuracy remain important tasks for future work.

## 4 Discussion

### 4.1 Alteration of FA during the Aging Process

Based on the above results, the alteration behavior during the aging process is discussed for FA1, FA2, and FA3 individually.

For FA1, the pH of the leachate reached approximately 12 immediately upon contact with water. The leaching concentrations of B and Se significantly decreased after the aging treatment, whereas the concentration of As was low even before aging. The aging treatment also led to a decrease in the leaching concentrations of Al, Ca, and SO_4_^2–^. SEM-EDS images showed roughening of the particle surface and the formation of needle-like precipitates composed of Ca and S due to aging. The MLA measurements showed no significant changes in the amounts of major compounds during the aging process, although a slight increase in the amount of ettringite was observed. These results suggest that contact with water immediately induces a highly alkaline condition in FA1. Under such pH conditions, a small amount of ettringite was formed, but the major components remained largely unchanged. Ettringite has been reported to immobilize toxic elements such as B, As, and Se,^37,46,54–56^ which is consistent with the results of this study. In addition, based on previous studies, trace amounts of Ca coprecipitates (Ca salts) with the toxic elements may have formed because FA1 is comparably rich in Ca;^46,57–59^ however, these were likely below the detection limit of the MLA measurement. In addition, As did not leach out even without the aging treatment despite its total content of 4.53 mg/kg, indicating that it had already been immobilized, possibly as a Ca-bearing compound.^60^

For FA2, the leachate was initially acidic (pH 4), with the pH increasing to approximately 10 over time. The aging treatment did not result in the immobilization of B, As, or Se. The concentration of Al increased in parallel with the increase in pH during the 6-h leaching experiment. After the aging treatment, the pH remained relatively stable at approximately 9 during the leaching period, and the concentration of Al decreased compared to that of the untreated sample. The leaching concentration of Ca increased with age, which is inconsistent with that in FA1. The MLA measurements showed an increase in the quartz content (pure SiO_2_), which was attributed to the dissolution of mullite and muscovite during aging. This trend was consistent with the Al leaching behavior observed for the untreated sample. These findings suggest that aluminosilicates (including K-aluminosilicate) dissolved under the initial acidic conditions and subsequently transformed into SiO_2_ during aging. Furthermore, the ettringite content did not increase based on the MLA results. This indicates that the formation of needle-like crystals, such as for ettringite, observed via SEM-EDS was likely limited. This also reconfirms the limitations of SEM-EDS analysis in characterizing samples such as FA, which exhibit significant particle-to-particle heterogeneity. The relatively low Ca content and pH of FA2 compared to those of FA1 also imply that ettringite or Ca salts with the toxic elements were not formed; thus, these toxic elements were not immobilized.

For FA3, the pH of the leachates was 9–10 (weakly alkaline). The aging process resulted in a slight decrease in the leaching of B and Se but an increase in the leaching of As. The leaching experiments showed a decrease in the concentrations of Al and Ca with time owing to aging. The SEM-EDS analysis indicated the formation of plate-like crystals composed of Ca and S during aging. The MLA results indicated that quartz and mullite increased while muscovite drastically decreased after aging. Additionally, ettringite increased after aging. Considering the pH range required for the stability of ettringite and the SEM-EDS observations, the increase in ettringite in FA3 may be attributed to the formation of monosulfate (kuzelite). Monosulfate has been reported to immobilize anions,^61–63^ suggesting that the aging treatment applied to FA3 led to the formation of monosulfate and contributed to the partial immobilization of toxic elements. However, because of the low content of Ca and other alkaline components, the pH did not reach highly alkaline levels, and coprecipitates of Ca with the toxic elements may not occur. These contrasting alteration behaviors among the samples can be explained by differences in pH, which were related to the contents of CaO and SO_3_.^64,65^

### 4.2 Application of MLA to Samples with Fine Particles or Complex Matrix

Previous studies have revealed that MLA is indispensable for mineral identification in ores and improves the efficiency of mineral processing.^29,66^ In this study, MLA was applied as a new approach for the bulk analysis of FA to elucidate the alteration behaviors induced by aging and simulate changes resulting from contact with water. MLA enabled the macro-scale analysis of tens of thousands of particles, and the GXMAP mode further facilitated the detailed quantification of the composition of each grain. The MLA results revealed the characteristics of both major and minor components in the three FA samples, as well as the alteration behaviors caused by aging. These findings demonstrate that MLA is promising not only for conventional ore analysis but also for the macro-level analysis of environmental samples with complex matrices, such as FA, soil, and cementitious materials. Although some issues remain to be addressed—such as refining sample pretreatment methods, improving quantification accuracy, and expanding the databases related to cementitious minerals, MLA applications are expected to expand to environmental samples in the future.

## 5 Conclusions

The alteration behaviors of FA1, FA2, and FA3 during the aging process varied significantly depending on their initial pH and compositional differences. Notably, comprehensive mineral quantification by MLA revealed the changes in major mineral phases and formation trends of minor secondary phases induced by the aging treatment. In FA1, a highly alkaline condition (pH 12) was rapidly induced upon contact with water, and MLA showed a slight increase in ettringite (Ca_6_Al_2_(SO_4_)_3_(OH)_12_•26H_2_O) content after the aging. In addition, the leaching concentrations of B and Se markedly decreased, suggesting the immobilization in Ca compounds such as ettringite or other Ca salts. In addition, As was immobilized even without aging, likely due to the presence of Ca-bearing compounds. In contrast, FA2 initially exhibited acidic conditions (pH 4), which gradually increased to pH 10 over time. The concentrations of B, As, and Se did not change due to aging, nor did the MLA indicate secondary formation of ettringite. Additionally, an increase in the amount of quartz was observed by MLA, suggesting the dissolution of aluminosilicates such as mullite and muscovite during aging at weakly acidic to neutral pH. Furthermore, FA3 exhibited weakly alkaline conditions (pH 9–10), and SEM-EDS and MLA indicated an increase in ettringite by the aging treatment. Considering the formation conditions of ettringite, the formation of monosulfate (kuzelite), Ca_4_Al_2_(OH)_12_(SO_4_)•6H_2_O, which has a similar composition to ettringite, was suggested for FA3, and this corresponded to a decrease in the leaching of B and Se. These results suggest that the immobilization behavior of toxic elements during the aging process is governed by the pH behavior and formation of secondary phases, as quantitatively revealed by the MLA. The application of the MLA has proven effective in identifying changes in both major and minor mineral phases, even in heterogeneous environmental materials such as FA.

## Supplementary Material



## Notes

(Figure S1) pH changes in the initial 40 min during the 6 h leaching experiment with time; (Figure S2) leaching behavior of major components (Si, Al, Ca, Na, K, Mg, and SO_4_^2–^) in the initial 40 min during the 6 h leaching experiment with time; (Figure S3) leaching behavior of toxic elements (B, As, and Se) in the initial 40 min during the 6 h leaching experiment with time; (Figure S4) SEM-EDS images of FA3, A-FA2, and A-FA3; (Figure S5) MLA mapping images of samples before and after the aging treatment (PDF)
