# Supplementary material for: Alteration of Coal Fly Ash Induced by Aging Treatment: Insights from Mineral Quantification Analysis
Source: ACS Omega. 2025 Oct 10;10(41):48787–97. doi: 10.1021/acsomega.5c06820 (PMC12547556; doi:10.1021/acsomega.5c06820)
Supplement: Supplementary file 1 [file ao5c06820_si_001.pdf]

Alteration of coal fly ash induced by aging treatment: insights from mineral quantification analysis

Tsugumi Seki[1, *], *Tatsuru Takahashi*[2], *Taiji Chida*[1], *Chihiro Inoue*[3], *Yasumasa Ogawa*[4]

[1] Department of Quantum Science and Energy Engineering, Graduate School of Engineering, Tohoku University, Aoba 6-6-01-2, Aramaki, Aoba, Sendai, 980-8579, Japan

[2] Seafloor Mineral Resources Department, Japan Organization for Metals and Energy Security, 2-10-1 Toranomon, Minato-ku, Tokyo, 105-0001, Japan

[3] Graduate School of Environmental Studies, Tohoku University, 6-6-20, Aoba, Aramaki, Aoba-ku, Sendai, 980-8579, Japan

[4] Department of Earth Resource Engineering and Environmental Science, Faculty of International Resource Sciences, Akita University, 1-1, Tegatagakuen-machi, Akita, 010-8502, Japan

KEYWORDS: coal fly ash, mineral liberation analysis, mineral classification, aging treatment, toxic elements

[*] tsugumi.seki.a5@tohoku.ac.jp

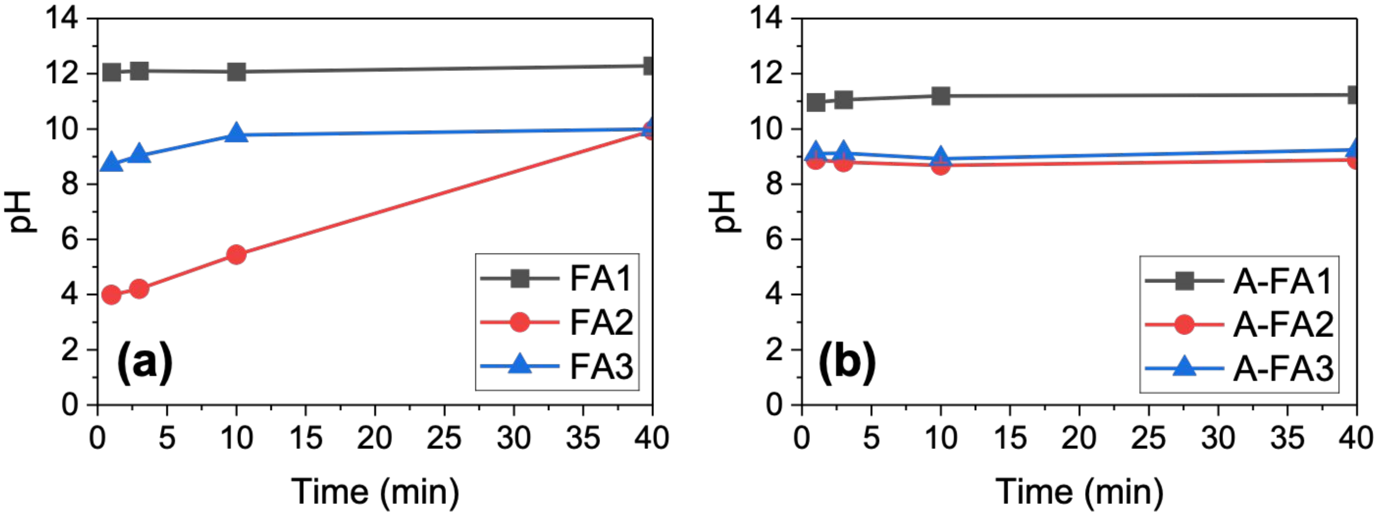

Figure S1 pH changes in the initial 40 minutes during the 6-hour leaching experiment with time. Error bars represent the standard error of triplicate experiments.

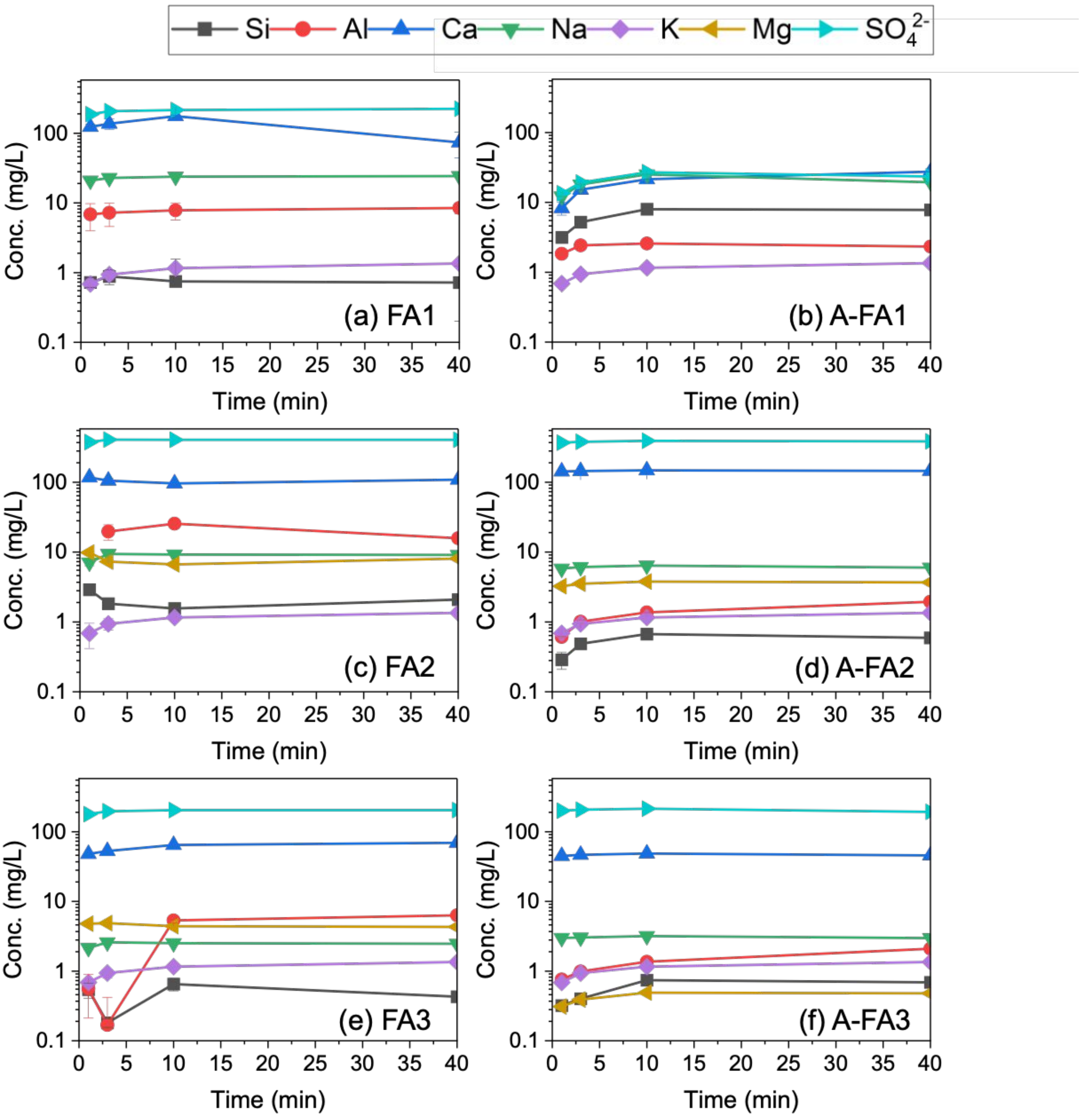

Figure S2 Leaching behavior of major components (Si, Al, Ca, Na, K, Mg, and SO_4^{2-}) in the initial 40 minutes during the 6-hour leaching experiment with time. Error bars represent the standard error of triplicate experiments.

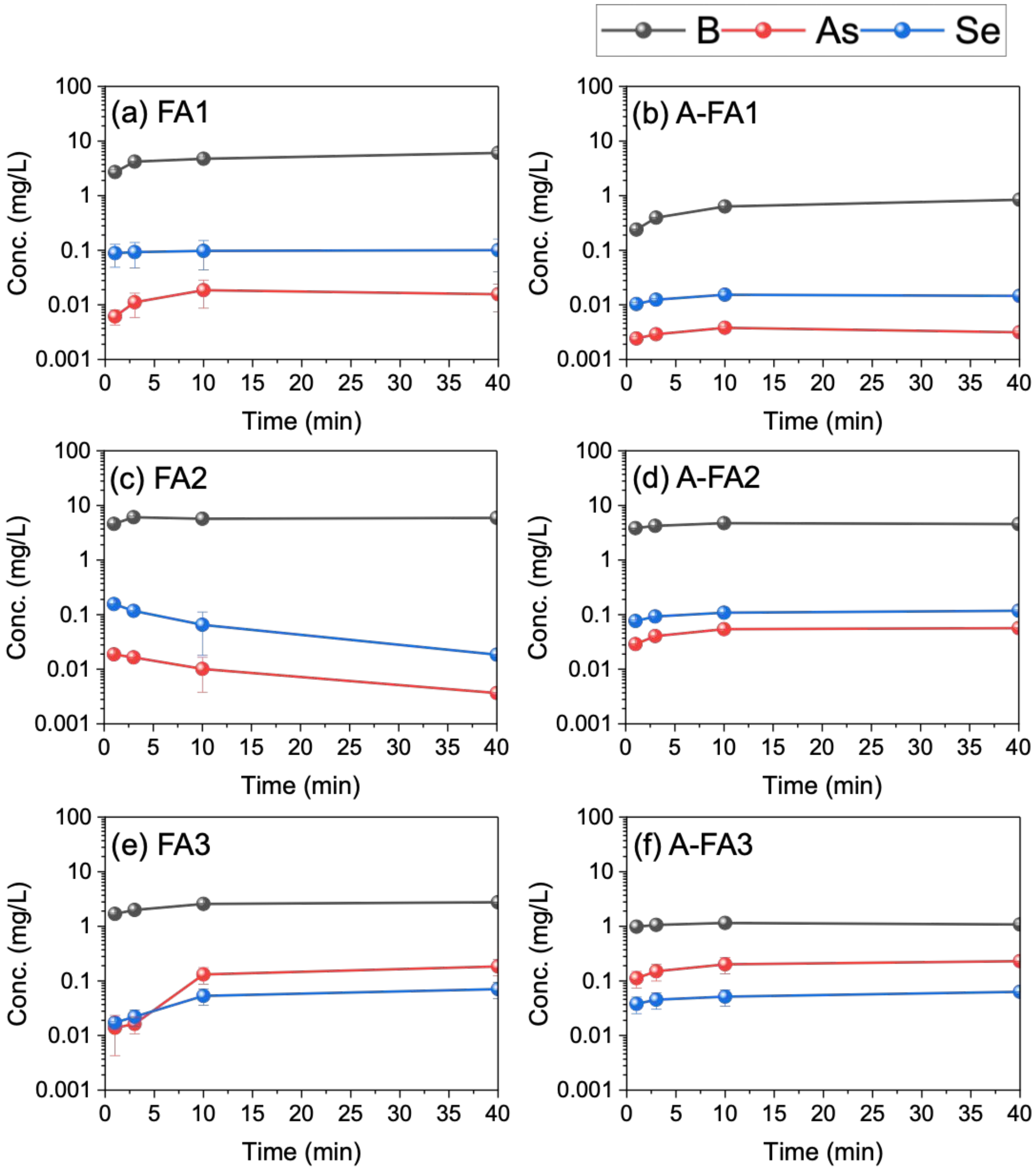

Figure S3 Leaching behavior of toxic elements (B, As, and Se) in the initial 40 minutes during the 6-hour leaching experiment with time. Error bars represent the standard error of triplicate experiments.

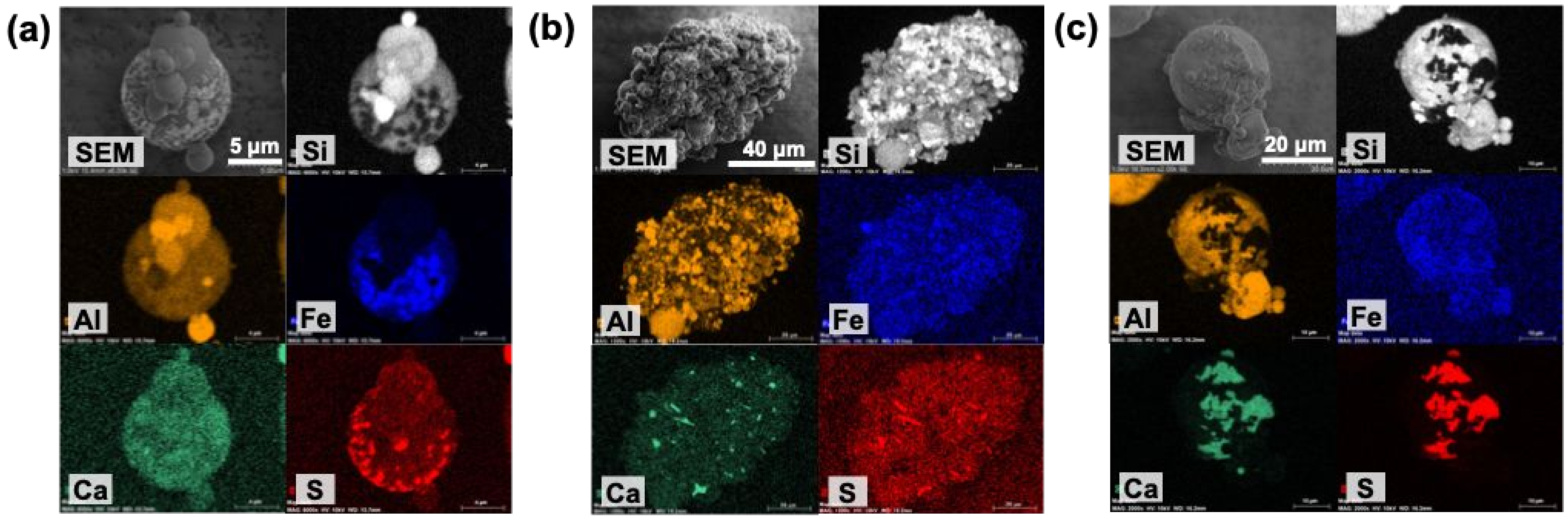

Figure S4 SEM-EDS images of (a) FA3, (b) A-FA2, and (c) A-FA3. In FA3(a), unevenly distributions of iron and sulfur was observed. In A-FA2 (b), particle aggregation was observed, along with partial needle-like crystals where Ca and S were detected. In A-FA3 (c), faceted tabular crystals with a distribution of Ca and S were observed.

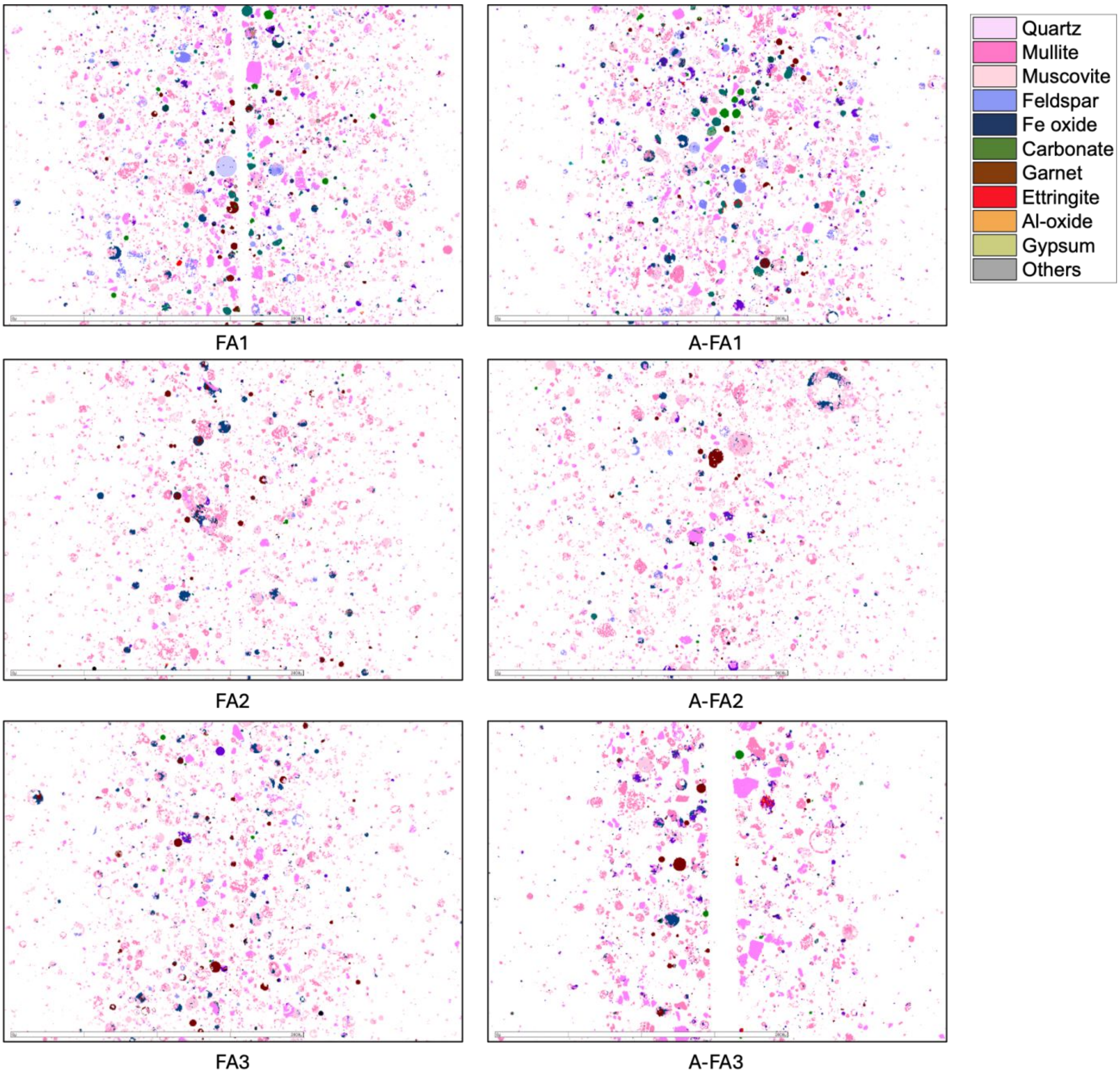

Figure S5 MLA mapping images of samples before and after the aging treatment.
